# Engineering Tocopherol Selectivity in α-TTP: A Combined *In Vitro/In Silico* Study

**DOI:** 10.1371/journal.pone.0049195

**Published:** 2012-11-13

**Authors:** Rachel E. Helbling, Walter Aeschimann, Fabio Simona, Achim Stocker, Michele Cascella

**Affiliations:** Department of Chemistry and Biochemistry, University of Bern, Bern, Switzerland; King’s College, London, United Kingdom

## Abstract

We present a combined *in vitro/in silico* study to determine the molecular origin of the selectivity of 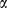-tocopherol transfer protein (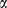-TTP) towards 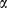-tocopherol. Molecular dynamics simulations combined to free energy perturbation calculations predict a binding free energy for 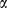-tocopherol to 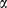-TTP 8.26
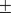
2.13 kcal mol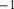 lower than that of 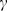-tocopherol. Our calculations show that 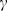-tocopherol binds to 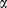-TTP in a significantly distorted geometry as compared to that of the natural ligand. Variations in the hydration of the binding pocket and in the protein structure are found as well. We propose a mutation, A156L, which significantly modifies the selectivity properties of 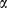-TTP towards the two tocopherols. In particular, our simulations predict that A156L binds preferentially to 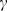-tocopherol, with striking structural similarities to the wild-type-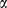-tocopherol complex. The affinity properties are confirmed by differential scanning fluorimetry as well as *in vitro* competitive binding assays. Our data indicate that residue A156 is at a critical position for determination of the selectivity of 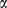-TTP. The engineering of TTP mutants with modulating binding properties can have potential impact at industrial level for easier purification of single tocopherols from vitamin E mixtures coming from natural oils or synthetic processes. Moreover, the identification of a 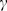-tocopherol selective TTP offers the possibility to challenge the hypotheses for the evolutionary development of a mechanism for 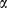-tocopherol selection in omnivorous animals.

## Introduction

Vitamin E is a generic term for substances known to be biologically and physiologically essential to health for their antioxidative properties in membranes [1], [2]. Each of the eight recognized natural vitamin E compounds, namely 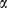-, 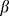-, 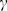-, and 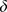-tocopherol and -tocotrienol, has the propensity to act as chain-breaking antioxidant in the chain reaction of lipid peroxidation. 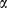-tocopherol (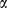-T hereafter) is the most important lipid soluble antioxidant in the body protecting cell components from oxidative damage [3]. Upon reaction with free radical species as well as with singlet oxygen, the vitamin may eventually undergo irreversible oxidation yielding mostly tocopheryl quinones [4]. In the human body, excess tocopherols and tocotrienols are metabolized to water-soluble carboxyethyl hydroxychroman metabolites and excreted in the urine [5]. It has been postulated that vitamin E may have roles in the human biological system other than that of an antioxidant molecule [6]. The ability of vitamin E to modulate signal transduction and gene expression has been observed in numerous studies, although the underlying molecular mechanisms have remained obscure. For example, at the transcriptional level, 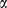-T modulates the expression of the CD36 scavenger receptor in smooth muscle cells and monocyte-derived macrophages, the hepatic 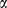-T transfer protein (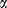-TTP) as well as the expression of liver collagen alpha-1, collagenase and the 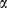-tropomyosin gene (for review see the work of Rimbach *et al.* in Ref. [7]). Low levels of 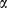-T are associated with neurological phenotypes in mammals, like human Ataxia with Vitamin E Deficiency (AVED) [8]–[11].

Tocopherols are quantitatively the major vitamers, whereas the tocotrienols are found *in vivo* at much lower concentrations. All tocopherol isoforms possess three chiral centers, so, in principle, eight diastereoisomers can be synthetically obtained and are widely used in animal nutrition [12] as well as in cosmetic products [13]. Currently, the tocopherol manufacturing implies the production of about 35,000 tons of racemic mixture per year [14]. The highest bioavailability is associated to the naturally occurring *RRR*-diastereoisomer of 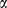-T [12], [15].

**Figure 1:**
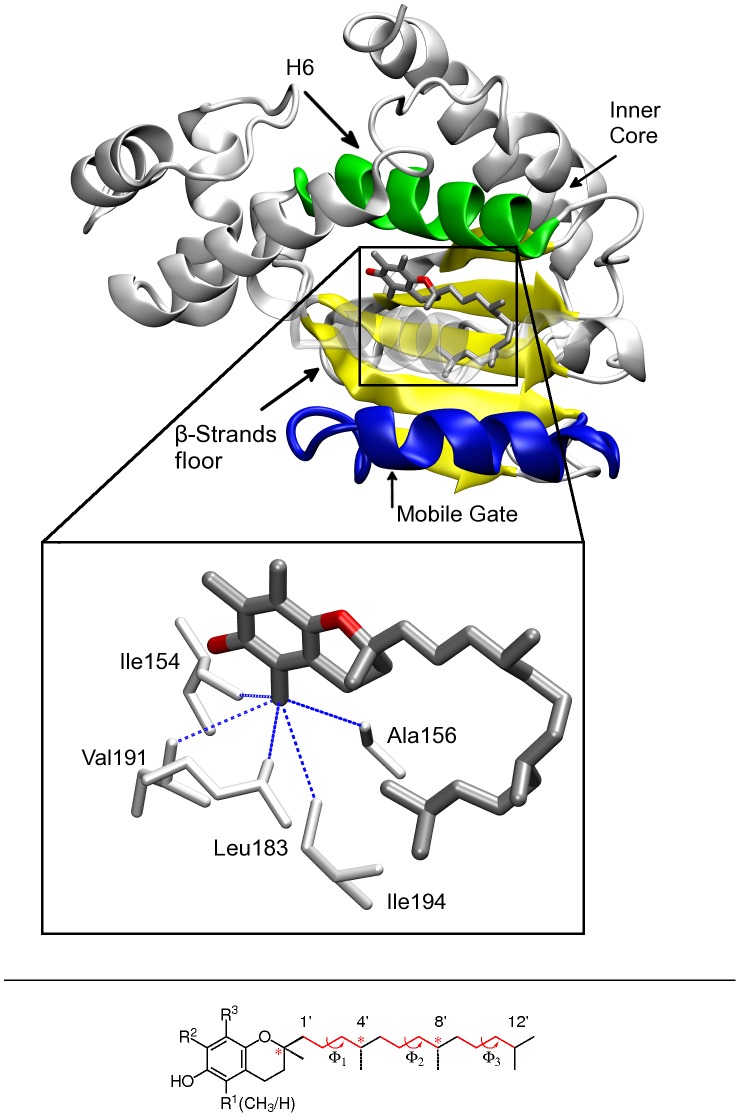
Structure of WT 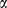
 **-TTP (from X-ray data)** [**9**] **, bound to** 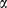 **-T.** *Top panel*, the four faces of the binding pocket are highlighted - the Helix 6 (H6) in green, the 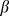-strand floor of the cavity in yellow, the mobile gate part in blue and the inner core of the binding pocket (the back part in the picture); *inset* 3D rendering of 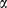-T bound to 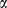-TTP. The residues in van der Waals contacts with the R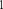 methyl group are highlighted in white licorice. *Bottom panel*, chemical structure of tocopherol. 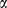-T: R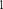 = R^2^ = R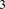 = CH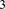; 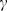-T: R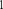 = H and R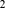 = R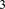 = CH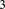. The chiral centers are highlighted by red asterisks.


The lipophilic vitamin E molecules require a specific cytosolic transfer protein, 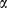-TTP, to facilitate their transport through hydrophilic media and to be assimilated by the organism. 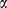-TTP is a 32 kDa protein, and was first described by Catignani in 1975 [16], [17]. It belongs to the Sec-14 like protein family, known to be involved in lipid regulation [18]. These proteins share a common CRAL-TRIO [19], [20] fold of approximately 185 amino acids. The fold consists of five parallel 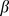-strands constituting the floor of the binding cavity, a variable number of 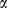-helices and a mobile helical gate at the carboxy-terminal region [21], [22] that allows the lipophilic ligand to access the binding pocket (Figure 1) [9], [21]. 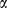-TTP has been isolated in both rats and humans, and it is mainly expressed in the liver, but it is also present in the placenta and in the brain [23]. 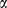-TTP plays a key role in the regulation of vitamin E in hepatocytes [24]. Correct expression of 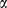-TPP is essential to the health of the organism, as its poor expression or mutation is directly associated to occurrence of AVED genetic disease. Recent work shows that the binding of 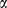-TTP is most favorable to small unilamellar vesicles, as observed by other membrane binding proteins [25].

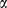
-TTP is responsible for the selection and retention of only the 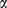-T vitamer in the body [26], [27]. Based on original crystallographic data, it has been postulated that the selectivity mechanism occurs via optimization of van der Waals contacts between 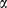-T and the surrounding protein environment at the chromanol ring site [9], [26], [28], [29]. Also, the lower binding affinity of other tocopherols presenting a lower number of methyl groups at the chromanol ring, like 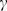-tocopherol (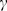-T), was putatively connected to a smaller tocopherol surface, which, in turn, would be less effective in forming hydrophobic interactions [30]. Nonetheless, a detailed picture of the molecular mechanism that regulates the selectivity of such protein towards 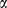-T are still not clear.

In this work, we report a combined *in vitro/in silico* investigation on the binding properties of 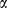-TTP towards 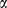-T and 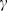-T (see Figure 1). Classical Molecular Dynamics (MD) simulations in combination with Free Energy Perturbation (FEP) methods [31]–[41] were used to investigate in detail both the energetic and structural features of binding of the substrate molecules to 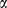-TTP. Our calculations are in very good agreement with *in vitro* data, and show that the mainly energetic contribution regulating the binding affinity comes from hydrophobic interactions; nonetheless, the protein conformational flexibility has a determining role in the relative stability of different tocopherols; in particular, we present one mutant that shows inverse selectivity towards 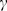-T and 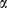-T, with respect to the wild-type protein (WT).

The newly found isoform is of great physiological interest, since 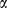-T may be involved in enzyme activation and gene regulation [42] while 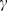-T, besides its antioxidant properties, shared with other isoforms, shows anticarcinogenic activity [43]. Design of TTP mutants able to selectively bind different forms of tocopherol is therefore of potential great interest for multiple purposes. In fact, these mutants could be of use for both follow up mutagenesis studies aiming at a comprehensive description of vitamin E function, and for purification protocols in tocopherol industrial processes.

## Results and Discussion

### Experimental Results

#### Competitive binding assays

Human 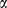-TTP genes (wild-type, A156L) were overproduced by heterologous expression in *E.coli* and their in vitro substrate specificity assessed in an aqueous micellar system including detergent solubilized tocopherols. For this purpose equimolar amounts of 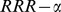
- and 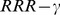-tocopherol were solubilized using a 50-fold excess of n-Octyl-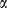-D-Glucopyranoside. Wild type 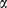-TTP and A156L were incubated in the presence of mixed micelles at a 66-fold molar excess of tocopherol. Detergent was subsequently removed by dialysis and monomeric tocopherol-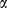-TTP ligand complexes were isolated by SEC chromatography, lyophilized and bound tocopherols analyzed by HPLC. For wild type 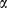-TTP a molar ratio of 5.3∶1 for 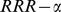-T/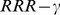-T was determined. The preferential binding of 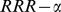-T relative to 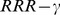-T for wild type 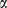-TTP confirms, though on a qualitative level, previous *in vitro* findings by the Hosomi group [27] where ligand specificity was assessed in a competitive assay using non-labeled tocopherol analogs and 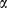-[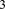
H]tocopherol for transfer between membranes. We have also determined binding specificities for the 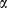-TTP mutant A156L yielding ratio of 1∶1.5 for 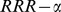-T/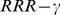-T respectively. Our results unequivocally indicate the successful functional modification of the ligand specificity of wild type 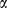-TTP towards increased 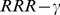-T selectivity for the A156L mutant. The combination of equilibrium dialysis and SEC chromatography offers a simple and reliable way for the production and analysis of tocopherol-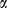-TTP ligand complexes at a preparative scale and may aid in further steps towards X-ray structural elucidation. Among a wide range of commercially available non-charged detergents n-Octyl-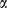-D-Glucopyranoside was selected due to its high tocopherol solubilizing capacity and its easy removal by dialysis. Our method may also be helpful for the comparison of binding data using non-natural tocopherol congeners and may aid to circumvent 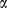-TTP's notorious propensity for multimer formation and aggregation.

#### Thermodynamic analysis of differential scanning fluorimetry data

In general, differential scanning fluorimetry (DSF) is a method which monitors thermal unfolding of proteins in presence of a fluorescent dye with a RT-PCR machine. There are several fluorophores which are reported to be used commonly in DSF experiments [44], [45]. In our case we used the thiol-reactive fluorescent dye BODIPY-FL L-Cystein (Invitrogen catalog no. B-20340). It is virtually nonfluorescent in its dimeric conformation. However, in complex with thiols a strong green fluorescence results. Thus upon interaction with emerging cysteins of denaturing proteins the fluorescence increases proportionally to the amount of available free cysteins. Hence, after defining a minimum (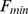) and maximum (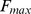) fluorescence it is possible to calculate the ratio of folded vs. unfolded protein at any temperature with equation 1.
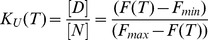
(1)


The temperature change can be related to the change in equilibrium by the van't Hoff equation (equation 2) giving the enthalpy 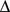
H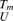 for the unfolding at the transition point. The natural logarithm of the equilibrium constant was plotted against the reciprocal temperature and a linear fit was performed. The fit gives a line with a slope equal to minus the standard enthalpy divided by the gas constant. This was done at eight different melting point for each protein sample; melting point shifts were induced by adding urea in increasing steps to the protein solution.
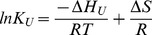
(2)


Since 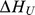 can be measured as a function of temperature it is possible with the Kirchoff equation (3) [46] to calculate the difference in heat capacity, 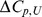 for the reaction folded vs. unfolded.
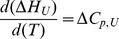
(3)


The temperature dependence of the free energy of unfolding 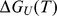 is described by the Gibbs-Helmholtz equation (4). With the enthalpy of unfolding 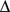
H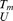, the melting temperature 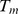 and the change in heat capacity upon unfolding 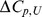 it is possible to plot 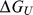 as function of temperature.
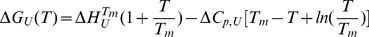
(4)


**Figure 2:**
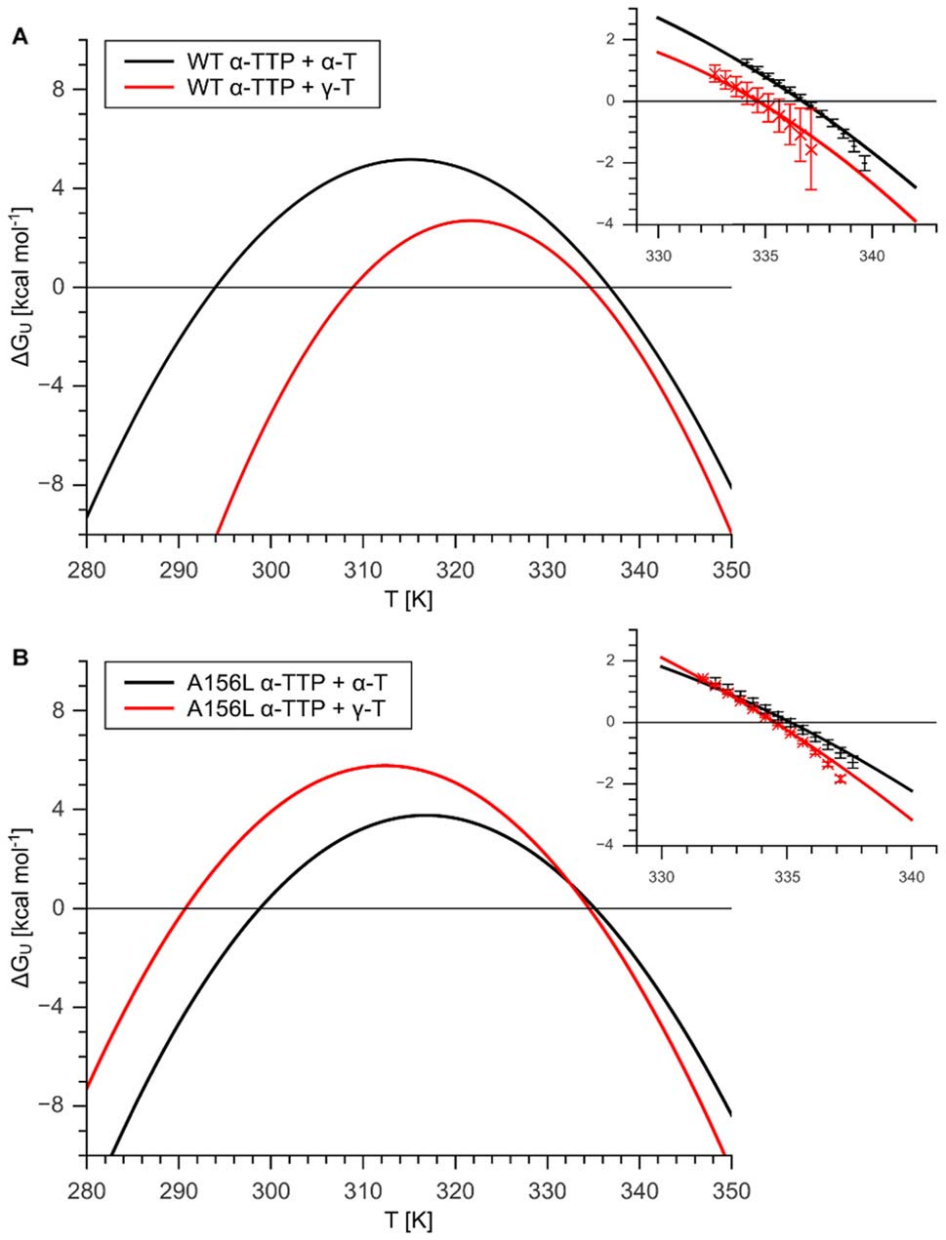
Gibbs-Helmholtz plots for 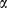
 **-TTP-tocopherol complexes.** Panel A shows 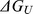 as a function of temperature calculated with equation (4) using data described in Table 1 for WT 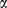-TTP in complex with 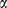-T and 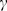-T respectively. Panel B shows the same for the A156L mutant in complex with tocopherols. In both panels a cutout around the melting point (T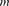) is shown, wherein calculated 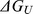 values and measured 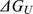 around the T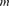 for the respective 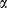-TTP are compared. The measured 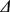
G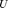 values are averages of replicate melting curves 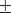 SEM.

The change in protein stability due to different ligands is equivalent to the free energy of binding to the native state if the ligand does not bind to the denatured protein [45] (see Table 1). Hence, the calculated free energies of a complex are sums of the free energy of unfolding (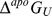) of the apo-protein plus the free energy of binding (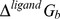) of the specific ligand. For quantifying the relative binding energies it is possible to subtract free energies of unfolding of different protein ligand complexes yielding a 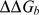 at any temperature. In our case we, 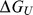 values of the protein complexed with 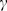-T were subtracted from the ones complexed with 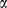-T; thus, a positive value indicates a higher affinity for 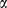-T, a negative value for 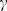-T. We report a 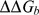 for WT of 7.67 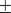 5.38 kcal mol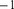 at 300 K and for A156L of −3.42 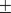 3.04 kcal mol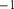 at 300 K. As shown in Table 2, the positive 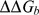 value of 7.67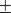 5.38 kcal mol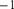 for WT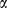-T is placed well between the value of 6.21 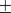 2.89 kcal mol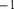 obtained by J. Atkinson and co-workers [22] the number obtained by our *in silico* calculations. In addition, our competitive binding experiment qualitatively confirms the high ligand specificity of the WT towards 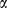-T. In the A156L mutant, for both the experimental approach as well as for the *in silico* calculations a negative  is obtained, indicative for higher affinity of A156L for 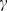-T than 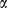-T. These results are again qualitatively confirmed by our competitive binding experiment. Though interpretations regarding thermodynamics need to be done with caution and have to be confirmed by other methods, one can rank very well compounds with similar physicochemical properties (e.g. 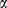-T vs. 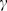-T) based on their relative 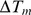 [47]. The high uncertainty of these results is a consequence of the calculus of the propagation of uncertainty. Since these values are calculated more than 30 K away from the melting points it is inevitable that the error is statistically high. However, our calculated data is in very good agreement with the measured data around the melting point (Figure 2), where it is possible to measure directly the 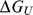 values from a melting curve.

**Table 1:** Thermodynamic parameters characterizing the unfolding of 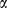-TTP complexes.

	T 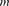 [K]	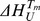 [kcal mol 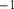 ]	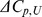 [kcal mol 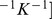 ]
WT 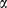 -T	336.73 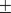 0.72	159.15 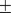 2.90	7.12 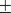 0.79
WT 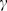 -T	334.65 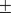 0.87	138.28 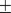 11.94	10.45 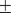 1.33
A156L 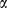 -T	335.15 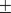 0.25	136.15 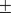 2.61	7.21 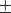 0.65
A156L 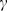 -T	334.53 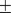 0.13	172.29 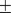 1.98	7.51 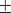 0.88

All data are denoted as the average 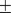 SEM, except for 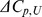 values where the slope of the weighted linear fit is the 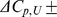 the error of the slope.

**Table 2:** Relative binding affinities (in kcal mol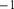) with respect to 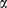-T.

Protein	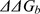 calcd	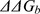 experiment	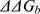 Previous data [22]	Comp. Binding Experiment
WT	8.26 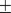 2.13	7.67 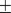 5.38	6.21 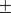 2.89	5.3∶1
A156L	−1.19 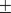 2.28	−3.42 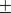 3.04	–	1∶1.5

Positive 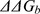 values indicate a preferred 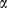-T binding. Calculated 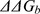 values were produced with equation 6 and experimental 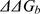 values with equation 4. The data indicate the mean values 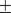 the standard deviation for both, experimental and computational data.

### Computational Results

#### Free energy of binding difference

Our FEP calculations estimate the relative binding affinity between WT 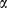-TTP and 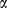-T or 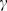-T in 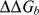  = 8.26 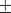 2.13 kcal mol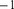. This computed data are in very good agreement with the experimental thermodynamic data obtained from DSF measurements as presented in Table 2, and with our competitive binding experiment which reports a 5.3∶1 preferential binding of 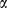-T to the WT than 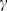-T.

FEP data indicate that A156L preferentially binds 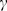-T than 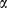-T (see Table 2). This mutation has particular relevance, as it corresponds to a functional modification of the WT. Corresponding competitive binding specificity experiments confirm this trend on a qualitative level.

The correct determination of the relative binding affinities in the WT indicates a very good reliability for the binding motifs found in our simulations for the various protein-ligand complexes. In fact, they all show subtle but significant differences at multiple contact sites between the proteins and the substrates from the WT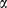-T natural template, which identification is crucial for the understanding of the origin of the selection mechanism.

#### Structural binding properties of 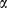-T and 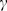-T in WT

In all our simulations, the RMSDs of the proteins C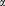 atoms are well converged after 100 ns (SI, Figure S1). Figure S2 in SI reports the average structures of the various protein-vitamin complexes after this simulation time.

**Figure 3:**
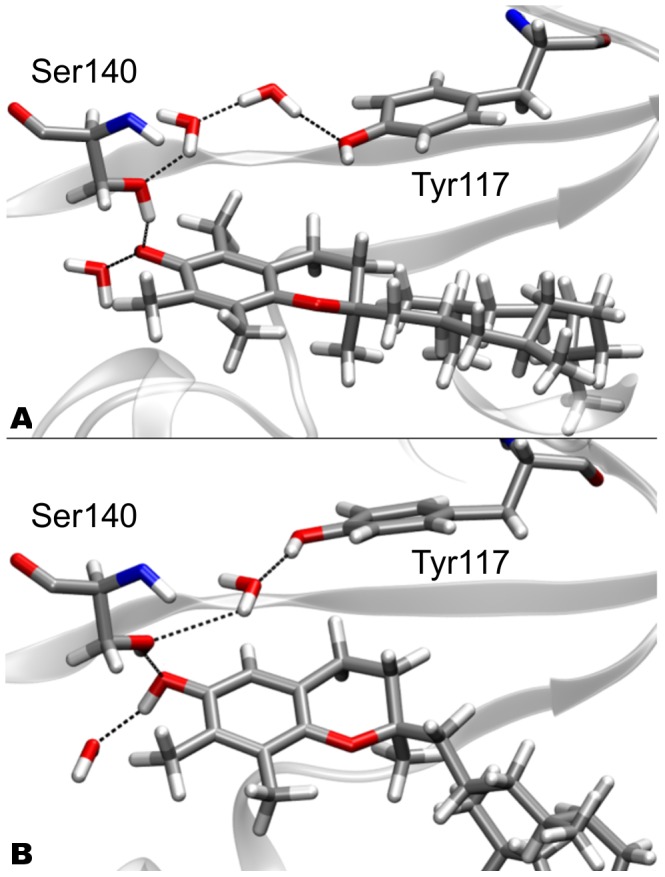
Hydrophilic interactions in the binding pocket of WT. Panel A (top): WT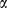-T; panel B (bottom): WT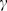-T. The residues involved in the H-bond network (Ser140 and Tyr117) are shown as licorice representation.

Vitamin E molecules have only one hydrophilic hydroxyl group, located at the 6 position of the chromanol ring benzene substituent, which is able to form H-bonds. The hydroxyl groups of both 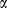-T and 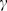-T form H-bonds with one crystallographic water molecule, and with the hydroxyl group of Ser140 (see Figure 3 and Table S1 in SI). X-ray diffraction of the WT [9], [28] shows the presence of three water molecules in the binding pocket next to the tocopherol hydroxyl group. These water molecules remain at the same location during simulations of the WT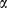-T complex. Two molecules are involved in a hydrogen bonding network between Tyr117 and Ser140. Strikingly, in the WT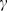-T complex, only one water molecule is present in this network (Figure 3), while the second molecule is not present the binding pocket, leaving it during the FEP transformation. The loss of one water is associated to a conformational rearrangement of the side chain of Tyr117 (hydroxyl oxygen distances between Tyr117 and Ser140 are 6.18 Å in WT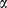-T and 5.50 Å in WT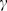-T), also occurring during the FEP.

**Figure 4:**
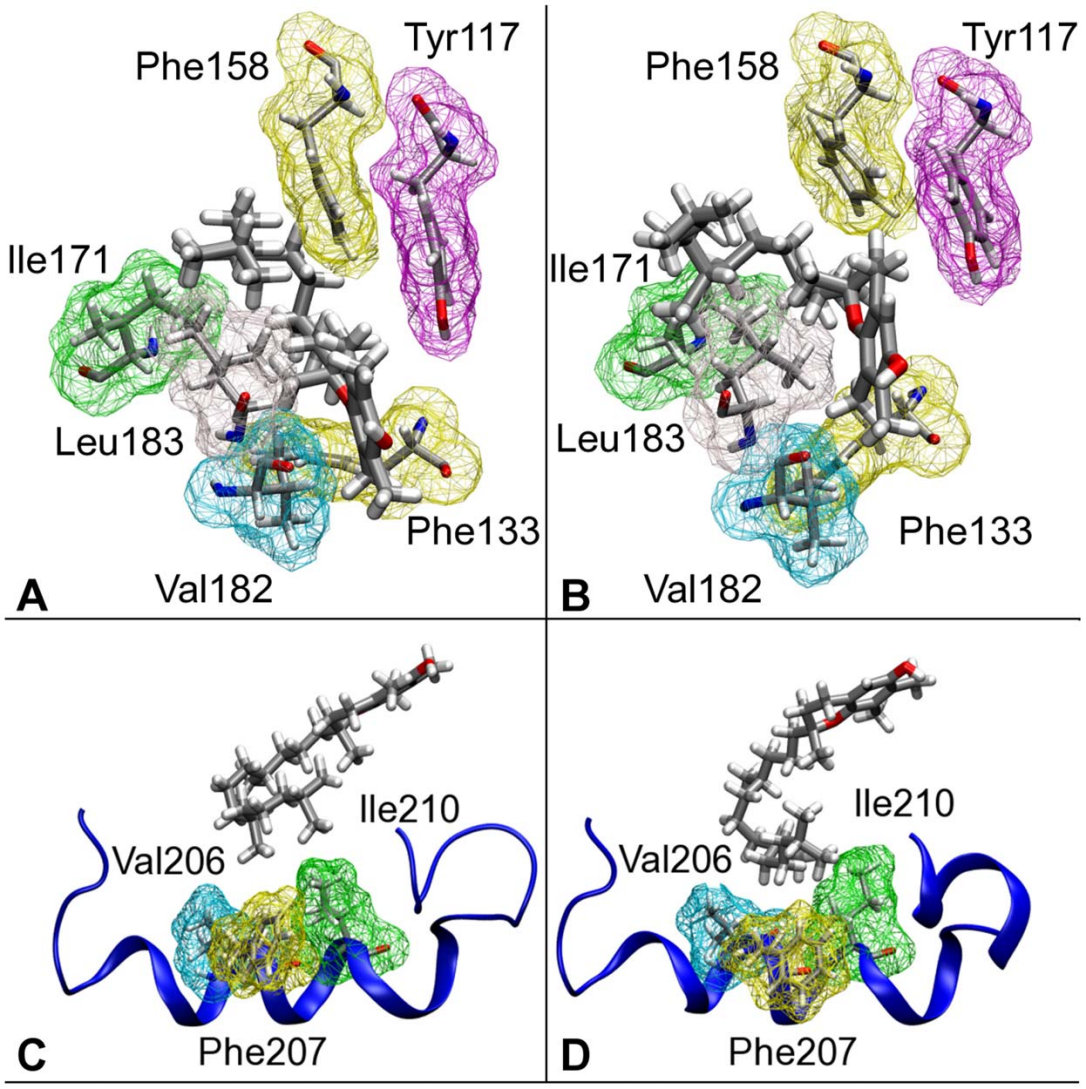
Comparison between hydrophobic contacts in WT 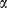
 **-T (left panels (A, and C)) and WT** 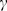 **-T (right panels (B, and D)).** Top panels (A, B): the van der Waals space occupied by residues in the binding pocket is highlighted by the wireframe representation. The different conformation of the hydrophobic tail in 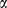-T and 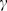-T is evidenced. Lower panels (C, D): comparison between the interaction of 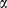-T (C) and 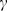-T with the helical mobile gate of the WT protein.

Significant differences in the position of the two tocopherol molecules in the binding pocket can be evidenced. In particular, the phtylyl tail of 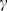-T assumes a different conformation than that of 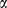-T. Also, in WT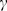-T, the chromanol ring is shifted towards the 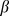-strands face of the binding pocket (Figures 3, and 4. For a conventional definition of the different sides of the binding pocket, refer to Figure 1). The van der Waals (vdW) contribution to the binding energy between protein and ligands was estimated by statistically averaging the vdW energy between the vitamin and the single protein residues present at the binding pocket along our simulations. The average vdW energies between each residue and the corresponding tocopherol molecule are listed in Table 3. Overall, the vdW contribution to the binding energy clearly favors 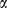-T than 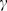-T. Differences in the interaction energies between 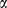-T and 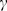-T and single residues are present, and can be associated to both the structural modifications in the ligand conformation and in the changes of shape of the protein binding pocket. 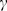-T is displaced towards the 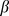-strand face of the binding pocket. As a result, Ile154, which is in contact with the R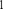 methyl group located at the 5-chromanol carbon in WT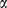-T, retains a similar vdW binding energy to both tocopherols (respectively −0.65 and −0.51 kcal mol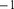), even though 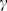-T lacks this methyl group. The maintainance of the contact between tocopherol and Ile154 is also evidenced by looking at the average distance between atoms CD1 of Ile154 and the C5 of the tocopherol, which is 5.26 Å and 5.34 Å for the WT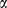-T and the WT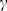-T, respectively (see SI, Figure S3 for the statistical distribution of the distances). The same behavior is observed for Leu183 and Ile194, also located in this region (Table 3). On the contrary, vdW contacts between 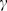-T and residues located at the H6 face of the binding pocket are partially lost. In particular, residues Trp122, Phe133 and Ser140 lose a total interaction energy of 2.20 kcal mol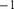. In this area, a partial rearrangement of the side-chains is observed. In particular, the side-chain of Phe133, facing the site of the C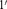 atom in the hydrophobic tail in the WT, presents a different 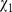 dihedral angle when 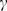-T is bound to 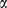-TTP compared to the WT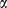-T complex (−107.63 in WT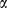-T, −173.86 in WT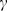-T complex; see Figure 4 and Table S2 in SI). Also, the average distances between the CE2 atom of the Phe residue and C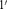 of the tocopherol are showing a slightly weaker interaction, with distances of 4.39 Å and 4.49 Å in WT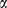-T and WT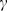-T, respectively (see SI, Figure S3). In this conformation, the side chain of Phe133 maintains the contact with the R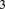 methyl group of the chromanol ring. This interaction is counterbalanced by the loss of a contact between the phenyl ring of Phe133 and the C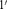 atom of the phtylyl tail of the tocopherol.

**Table 3:** Comparison between van der Waals interactions of the different studied systems.

Residue	WT 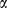 -T	WT 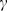 -T	A156L 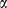 -T	A156L 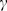 -T
Tyr117	−2.21	−2.52	−2.08	−1.24
Trp122	−1.09	−0.78	−0.72	−0.90
Phe133	−5.13	−3.15	−5.32	−5.12
Ser140	−0.42	−0.51	−0.08	−0.90
Ile154	−0.65	−0.51	−0.57	−0.66
Phe158	−4.15	−3.41	−4.35	−4.66
Trp163	−1.28	−1.30	−1.41	−1.28
Ile171	−1.60	−1.97	−1.42	−1.26
Ile179	−4.87	−4.41	−4.34	−4.90
Val182	−3.49	−1.72	−3.77	−3.57
Leu183	−3.30	−3.52	−3.09	−3.27
Phe187	−2.08	−1.26	−2.16	−1.98
Leu189	−0.73	−0.54	−0.66	−0.73
Ile194	−1.21	−1.52	−1.31	−1.20
Ile210	−1.42	−1.72	−1.49	−1.17
Phe203	−1.36	−1.37	−1.13	−1.45
Val206	−0.76	−0.93	−1.01	−0.48
Phe207	−0.54	−0.78	−0.89	−0.79
Ile210	−1.42	−1.72	−1.49	−1.17
Leu214	−0.35	−0.37	−0.41	−0.10
Leu218	−0.03	−0.08	−0.02	−0.01
Total	−38.09	−34.09	−37.72	−38.76

The van der Waals interactions (in kcal mol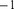) shown are between the tocopherol isoform and the binding pocket residues (upper part), and the most significant mobile gate residues (lower part).

At the mobile gate face of the binding pocket, the different binding geometry of 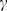-T is responsible for a weakening of the interactions between the aromatic part of the chromanol ring and the side-chain of Val182 (average distances between atoms CG2 of the residue and C8 of the tocopherol are 5.1 Å in WT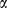-T and 5.9 Å WT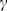-T, see SI, Figure S3). This loss of contact is counterbalanced by increased interaction with the side-chain of Leu183. The hydrophobic tail of 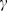-T makes different contacts with the protein with respect to those present in the WT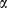-T complex. In particular, the interactions between the tail and Val206, Phe207, and Ile210, all residues belonging to the mobile gate segment, are modified (Figure 4).

The interaction between those residues and the terminal part of the hydrophobic tail is increased in WT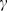-T. In particular, Ile210 interacts with the isopropyl end of the tocopherol in the WT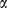-T, whereas a stronger contact with the same residue and the methyl group of C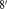 is observed. In addition, an increased interaction between Phe207 and the isopropyl end of the tocopherol tail in WT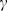-T induces a local structural modification of the C-terminal part of mobile gate segment, compared to the WT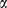-T system (Relative RMSD of the mobile gate segment: 1.32 Å). Different conformations in the side-chains of these residues are observed, leading to increased vdW interaction with 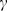-T, compared to the WT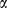-T system (Table 3, and Table S3).

The local distortions in the binding pocket occurring upon 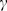-T binding also induce long-range modifications of the protein at its surface. In particular, helices H4, H5 are deformed, and partially lose their helical character. This portion of the protein shows a RMSD of 2.64 Å from the corresponding one in WT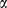-T, which is significantly higher than the average RMSD between the global structures of the two complexes (RMSD  = 1.63 Å).

#### A156L mutant

**Figure 5:**
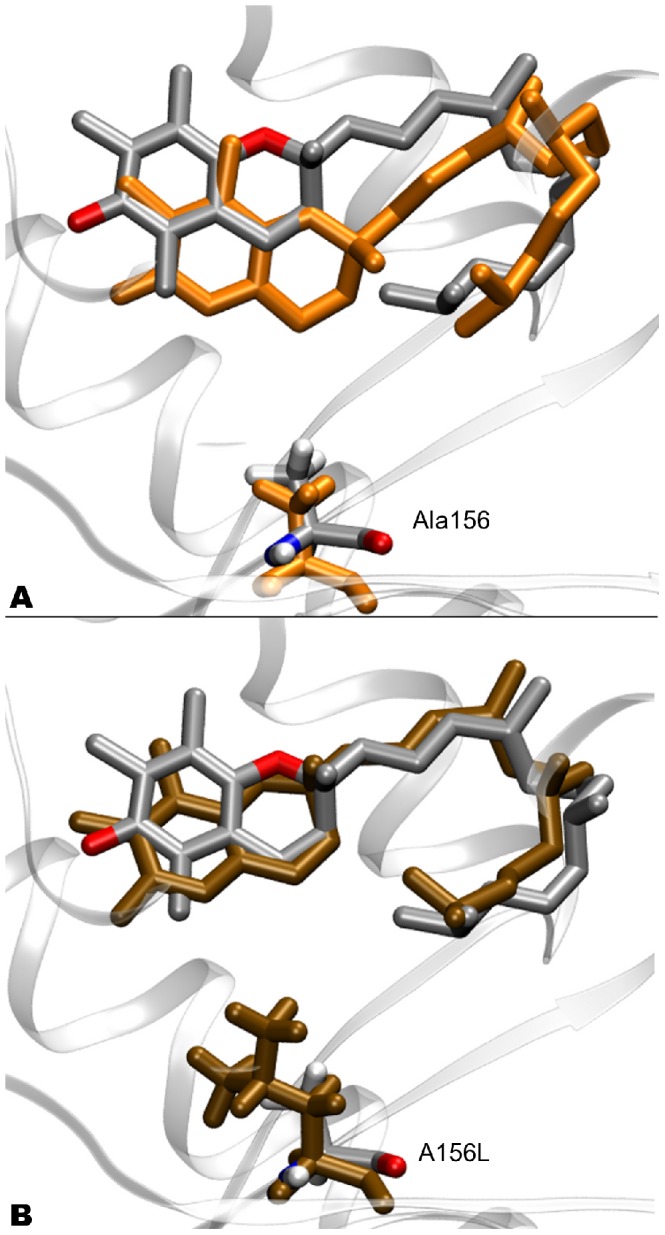
Comparison of tocopherol binding geometries in WT and A156L. Panel A represents WT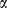-T compared to WT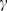-T (in ochre), the shift of the chromanol ring is evidenced. In panel B, comparison between WT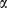-T and A156L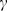-T (in ochre) is shown. The original position of the chromanol ring in the wild-type is retrieved for 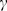-T. Comparisons are done between average structures from MD simulations. The position 156 is highlighted in every system. For clarity, the hydrogen atoms of the ligands are not represented.

The major structural distortion in WT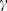-T is associated to the displacement of the cromanol ring toward a hydrophobic pocket formed by Ile154, Ala156, Leu183, Val191, and Ile194. Crystallographic contacts are highlighted in Figure 1. As the side-chain of Ala156 sits in this critical position (Figure 5, left panel), we hypothesized the possibility of mutating Ala156 into a bulkier hydrophobic residue, which would eventually lead to the back-displacement of 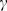-T into the original position observed for 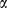-T in the wild-type protein (Figure 5, left panel).

Both calculations and experiment indicate a preferential binding for 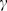-T than 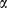-T in the A156L mutant. Despite the relative large error of both measurements, intrinsic to the methods used, the qualitative trend indicating a higher affinity for the 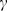 isoform of this mutant is confirmed by our 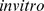 competitive assays (see Table 2). As originally postulated, the structural and dynamical properties of the A156L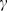-T complex have striking similarities to the ones found in the WT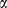-T one. The position of 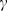-T in the binding pocket of A156L is very close to that of 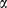-T in the WT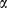-T structure (RMSD  = 1.49 Å, see Figure 5). In particular, the conformation of the phtylyl tail is significantly less distorted with respect to WT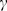-T, and the chromanol ring is shifted back towards the H6 side of the binding pocket.

The similarity in the binding modes of WT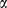-T and A156L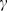-T is associated to the recovery of the vdW contacts between the ligand and the protein. In fact, the vdW energies between 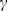-T and both Phe133 and Val182, which were significantly reduced in the WT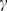-T complex, retain values similar to those of WT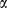-T in A156L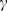-T (Table 3). The shift of the chromanol ring towards the H6 side is reflected by increased contacts between 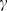-T and Phe133 and Ser140 residues, partly counterbalanced by an energy loss in the contacts formed by 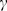-T and the Tyr117, and Ile194 residues belonging to the 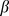-sheet region with respect to the same in the WT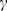-T complex. The contacts with the mobile gate helix are overall maintained, apart from slight modification in the reported vdW energies, which fall anyway within the statistical uncertainty. On a global scale, A156L binds with stronger vdW energy 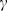-T than 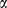-T (Table 3, and Figures S2 and S3).

The A156L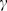-T complex shows significant similarities with the WT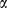-T one also in the hydration of the binding pocket. In fact, we find three buried water molecules, unlike in WT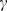-T, where only two molecules are present. Specifically, the two waters bridging the side-chains of Tyr117 and Ser140 are retained. The most significant difference between the hydration patterns in WT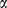-T and A156L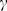-T lies in the observation that 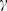-T is not directly hydrogen-bonded to Ser140, but it is connected to its side chain through one bridging water (SI, Figure S4). The hydration pattern of WT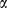-T complex is retained in A156L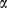-T. Nonetheless, in the two structures, the side-chain of Tyr117 assumes different conformations. In fact, its 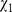 dihedral angle presents a different conformation in both systems, as shown by values of 93.37 in the A156L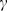-T complex, and −66.67 in A156L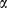-T. As a result, the distance between the hydroxyl oxygen atoms of Tyr117 and Ser140 differs from 6.39 Å in A156L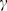-T (similar to the distance in WT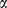-T), to 5.80 Å of A156L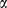-T. In A156L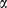-T all H-bonds present in the binding pocket are shortened; in particular, the H-bond between Ser140 and 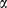-T shows a value of 2.17 Å in WT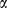-T and 1.84 Å in A156L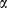-T.

Finally, in A156L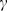-T the helices H4 and H5 do not show significant distortion. On the contrary, this region is deformed in the A156L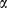-T complex, where a RMSD of 1.81 Å towards WT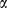-T, lower than the average RMSD for the two systems is found (RMSD  = 2.18 Å). In any case, the protein segment retains the helical structure, showing overall a minor distortion compared with that of the WT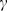-T complex. (SI, Figure S5).

## Concluding Remarks


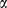
-TTP is a flexible protein, able to reshape its binding pocket to best accommodate different tocopherol ligands. The balance between formation of hydrophobic contacts and mechanical strain is responsible for determination of binding affinity between the protein and the ligand, and thus, responsible for the mechanism of selectivity of WT 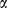-TTP towards 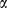-T. In addition, the water network and the hydrogen bonding play a key role in the stability and the positioning of the tocopherol within the binding pocket. The plasticity of 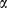-TTP can be used to design mutants that can modulate and even modify the natural function. In fact, we provide here for the first time *in vitro/in silico* evidence for the successful production of a functional 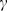-T selective TTP variant. In our corresponding experiment, the A156L mutant evidences a clear selectivity *in vitro* for 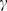-T: our calculations show that A156L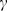-T complex retains the same structural properties of the WT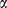-T.

Our data indicate the residue A156 as a critical position for the selectivity of 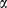-TTP. This evidence opens to the possibility of engineering other mutants, with modulated affinities for the different isoforms of vitamin E. The engineering of TTP mutants may have impact at industrial level for easier purification of single tocopherols from mixtures coming from natural oils or synthetic processes [14], [48].

Finally, identification of a 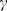-T selective TTP for the first time offers the possibility to challenge the 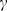-T competitive exclusion hypothesis *in vivo*. In fact, predicting and designing 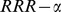-T mutants with high selectivity towards single tocopherol isomers demonstrates the great potential of mutagenesis for future studies aiming at a comprehensive description of vitamin E function. During evolution WT 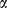-TTP has selectively acquired high affinity to 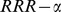-T. Accordingly, there must exist a genetic trait selection in favor of this tocopherol congener which is linked to a healthy phenotype. This implicates that competitive exclusion of 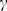-T in favor of 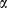-T must be advantageous for omnivores even in environments, where 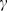-T is the principal dietary vitamin E source. Possible explanations for this phenomenon have been provided by Cornwell 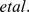 [49] suggesting that arylating quinones, including the partially substituted oxidized vitamin E congener 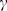-tocopherol quinone, effect ER stress and are cytotoxic, whereas the fully substituted nonarylating vitamin E congener 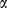-tocopherol quinone is not. Thus, retention of the nonarylating quinone precursor 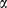-T by 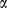-TTP possibly confers evolutionary benefits in animal cells and enhances protection against ER stress mediated pathogenesis such as type 2 diabetes [50], Parkinson's disease [51] and amyloid-beta neurotoxicity [52].

## Materials and Methods

### Experimental Setup

#### Protein overproduction and purification

TTP genes (wild-type, A156L) were synthesized at GeneArt and subcloned into Nde1 and Xho1 restriction sites of pET28 (Novagen). All three genes contained an N-terminal 6xHis-tag. Overproduction was carried out in 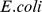 BL21 STAR under control of the T7 promoter by overnight induction using 100 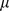
M isopropyl-thiogalactopyranoside at 37°C. Cells were harvested by centrifugation at 5000 rpm for 30 min and resuspended in 25 ml lysis buffer (20 mM Tris, 100 mM NaCl, 10 mM Imidazole, 0.8 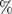 Triton X-100 and 1 mM PMSF). The cells were disrupted twice in a French press. Subsequently the cell suspension was centrifuged for 40 min at 16000 rpm and 4°C. Thereafter, the supernatant was pooled and applied to a Ni-NTA column (12 ml) on a Pharmacia FPLC system. Nonspecifically bound protein was removed with washing buffer (20 mM Tris, 100 mM NaCl, 40 mM imidazole, pH 8.0) until the UV absorption at 280 nm recovered the base line level. The protein was collected in elution buffer (20 mM Tris, 100 mM NaCl, 200 mM Imidazole, pH 8.0) in a final volume of 25 ml at a protein concentration of 0.5 mg/ml.

#### Tocopherol-specificity assay

Equimolar 1∶1 tocopherol mixtures were produced by overlaying 146.2 mg of n-Octyl-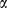-D-Glucopyranoside with 4.3 mg of 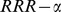-T and 4.2 mg 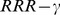-T respectively. The mixtures were centrifuged at 16'000 g for 5 minutes in order to create an oil in detergent matrix and then supplemented with 1 ml of Tris buffer (20 mM Tris, 100 mM NaCl, pH 8.0). The opaque tocopherol detergent solution (500 mM) was briefly vortexed and sonicated in a water bath until transparent. Subsequently 4.5 ml of 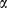-TTP protein solution were supplemented with 0.5 ml −/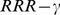-T mix and dialyzed against Tris buffer 4 times for 4 hours. The dialyzed protein solution was concentrated to 1 ml and purified by size exclusion chromatography (SEC). The fractions containing the monomeric ligand-protein complexes were pooled and lyophilized.

#### HPLC

Prior to HPLC analysis the lyophylized samples were dissolved in 80 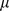
l methanol and shortly centrifuged at 18'000 g. HPLC analysis was carried out using a custom build JASCO HPLC (PU-980 pumps, UV-975 UV-detector and a Shimadzu C-R3A chart recorder). Full separation of tocopherol-ligands was accomplished on a reversed phase Waters Atlantis dC18 Column (3×100 mm, 5 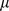
m particle size) and isocratic elution (mobile phase: 95 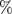 MeOH/5 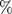 H2O) at a flow rate of 1 ml min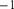. The effluent was monitored at 295 nm on the UV-detector and the absorbance was integrated with a Shimadzu C-R3A chart recorder. All injections to the HPLC were carried out with Hamilton syringes by injecting 5 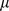
l of sample.

#### Determination of Thermodynamic Parameters by differential scanning fluorimetry


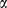
-TTP's final concentration was always kept at 7.5 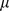
M. Protein concentration was determined by absorbancy at 280 nm using an extinction coefficient of 39545 M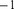 cm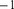. To take measurements, the protein solution was supplemented with fluorescent dye before being mixed with any additive. It was always added an equimolar amount of fluorescent dye (stock was 25.37 mM in DMSO) as the molarity of cysteins present in the protein of interest. Experiments were conducted in 10 mM Tris and 100 mM NaCl at pH 8.0; urea was supplement to achieve final concentrations in steps from 0.25 M, 0.5 M, 1M, 1.5 M, 2M, 2.5 M to 3M. Four 25 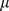
l replicas of each sample were measured on a 96-well plate with BioRad CFX 96 RT-PCR machine. Fluorescence was measured with filters to excite between 450–490 nm and measure emission between 515–530 nm. Unfolding of the protein was induced by a temperature gradient ranging from 20°C to 99°C with ramp of 1°C/min. Melting curves and inverse derivate curves were exported directly from the machine and calculations for thermodynamic parameters were performed using the van't Hoff equation. Replicate T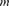 and 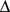
H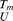 values were averaged and used for further calculations.

### Computational Methods

#### System setup

The starting structure of the wild type 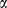-TTP (WT) bound to 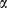-T was taken from the protein data bank (PDB entry: 1OIP [9]). Titrable groups were protonated at standard positions at pH = 7. The AMBER FF99SB [53] and the General Amber Force Field (GAFF) [54] force fields were used to parametrize the protein and vitamins, respectively. The RESP charge fitting procedure [55] was used to get the atomic charges of the tocopherols matching *ab initio* calculations at the B3LYP [56] level of theory (6–31G** basis set) using the GAUSSIAN 03 package [57]. The system was solvated with 19358 TIP3P water molecules [58] and one chlorine anion was added to achieve neutrality. We obtain a system formed by 62268 atoms with a box of 83.6×90.6×82.4 Å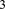. The same procedure was followed to setup a system containing 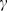-T bound to WT.

Models of the single-mutant proteins A156L were built by molecular replacement starting from the WT crystal structure. Simulations of the systems with both 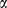
- or 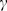-T bound to mutant proteins were prepared following the same procedure described for the WT.

#### MD simulations

A total of four systems were simulated: the WT protein, and its A156L mutant form, bound to either 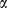-T or 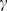-T. The structures were originally relaxed by 300 cycles of steepest descent minimization followed by 7200 cycles of conjugate gradients. A 100 ps run at constant temperature (300 K) and pressure (1 bar) keeping the protein immobile was performed to reach the correct density of liquid water, and then the system was minimized again. Nosé-Hoover thermostats [59]–[61] as well as Parrinello-Rahman barostats [62] were used to keep the system at constant temperature and pressure. The Particle-Mesh Ewald (PME) method [63] was used to treat the long-range electrostatic interactions with a cutoff of 12 Å, the non-bonded list was updated every 25 steps. The LINCS algorithm [64], [65] was used to constrain bonds involving hydrogen atoms. A time step of 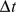  = 1.5 fs was used. The relaxed structures of all the systems considered were used as starting points for 100 ns long MD simulations.

#### Free energy perturbation

Free-Energy-Pertubation [31] calculations were used to estimate the relative binding affinities for 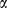-T and 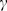-T in both WT and A156L. For this purpose, a coupling Hamiltonian, defined in equation 5 was used:
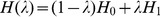
(5)where 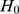 is the hamiltonian related to the system containing 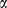-T, and 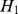 refers to the system with 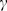-T. In particular, starting from 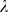 = 0 for a system comprising 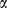-T (methyl group at the 5 position of the chromanol ring of tocopherol), 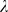 can be switched in small steps to 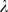  = 1, performing an alchemical modification of 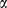-T to 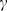-T (H atom at the 5 position of the chromanol ring). Using the Thermodynamic Integration formula [31]:



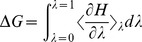
(6)the free energy difference between both states is obtained. In this work, the relative binding affinity (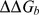) of the WT and relative mutants for the two tocopherols is computed. Following a standard thermodynamic cycle (SI, Figure S6), 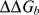 is obtained through the binding energy difference of the two ligands, in water and in the protein:



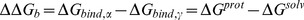
(7)For this purpose, the 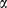-T to 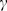-T alchemical reaction was computed, both in water (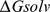) and in the protein environment (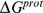).

Backwards transformations were also performed to balance hysteresis bias. Dummy atoms where used to keep a constant total number of particles along the transformation. The dual topology scheme was employed [31], [66], [67]. For each transformation, we used 7 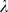-points. 7500 cycles of L-BFGS minimization [68], [69] were performed at the beginning of simulations at each 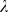 value, followed by 15 ns of FEP production at NpT conditions.

Structural analyses of the trajectories were performed with the VMD visualization software [70]. MD calculations and data analysis were performed with the GROMACS 4.0 package [71]–[74].

## Supporting Information

Figure S1
**RMSD of the C** 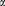 **atoms of the different complexes during the 100 ns of MD simulations.**
(TIFF)

Figure S2
**Average structures of topocherol in the studied complexes.** Panel A: WT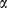-T; Panel B: WT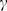-T; Panel C: A156L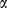-T; Panel D: A156L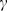-T.(TIFF)

Figure S3
**Statistical distribution of selected interatomic distances between tocopherol and residues in the binding pocket for the WT** 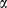 **-T and WT** 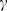 **-T complexes.**
(TIFF)

Figure S4
**Ligand-protein hydrophobic contacts in WT** 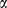 **-T (panel A) and 156L** 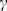 **-T (panel B).** The van der Waals space of residues in contact with tocopherol is highlighted by wireframe representation.(TIFF)

Figure S5
**Comparison of the structure of the H4-H5 segment for WT** 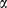 **-T (in red), WT** 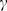 **-T (violet) and A156L** 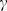 **-T (green) complexes.** The respective distribution of the amino acids in the Ramachandran plot is shown in the bottom panels.(TIFF)

Figure S6
**Scheme of the thermodynamic cycle used to compute the relative binding affinity of** 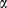 **-T and** 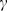 **-T to TTP.** Free-energy perturbation is used to estimate 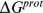 and 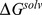.(TIFF)

Table S1 **Average H-bonding distances (in Å ) between the chromanol hydroxyl group of tocopherol and surrounding partners in each system.**
(PDF)

Table S2 **Comparison between specific dihedral angles of the hydrophobic tail of tocopherol in differerent tocopherol-TTP complexes.** The dihedral angles under consideration are highlighted in the bottom scheme.
(PDF)

Table S3
**Comparison between average values of the side-chain dihedral angles of residues in the binding pocket (top part) and relevant residues of the lid (bottom part) for the different complexes under study.** The 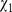 dihedral angle is the one corresponding to the rotation aroun the 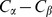 bond; the 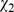 dihedral angle is defined by the 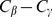 bond.(PDF)

## References

[1] Epstein S, Forsyth J, Saporosc IB, Mantel N (1966) An exploratory investigation on inhibition of selected photosensitizers by agents of varying antioxidant activity. Radiat Res 28: 322–335. 5941157

[2] Tappel A (1962) Vitamin-E as the biological lipid antioxidant. Vitam Horm 20: 493–510.

[3] Traber MG, Atkinson J (2007) Vitamin E, antioxidant and nothing more. Free Radic Biol Med 43: 4–15. 17561088 10.1016/j.freeradbiomed.2007.03.024 PMC2040110

[4] Thornton DE, Jones KH, Jiang Z, Zhang H, Liu G, et al (1995) Antioxidant and cytotoxic tocopheryl quinones in normal and cancer cells. Free Radic Biol Med 18: 963–976. 7628732 10.1016/0891-5849(94)00210-b

[5] Brigelius-Flohe R (2003) Vitamin E and drug metabolism. Biochem Biophys Res Commun 305: 737–740. 12763054 10.1016/s0006-291x(03)00811-8

[6] Packer L, Weber S, Rimbach G (2001) Molecular aspects of alpha-tocotrienol antioxidant action and cell signalling. J Nutr 131: 369S–373S. 11160563 10.1093/jn/131.2.369S

[7] Rimbach G, Minihane A, Majewicz J, Fischer A, Pallauf J, et al (2002) Regulation of cell signalling by vitamin E. Proc Nutr Soc. 61: 415–425. 10.1079/pns2002183 12691170

[8] Ouahchi K, Arita M, Kayden H, Hentati F, Benhamida M, et al (1995) Ataxia with isolated vitamin-E deficiency is caused by mutations in the alpha.tocopherol transfer protein. Nat Genet 9: 141–145. 7719340 10.1038/ng0295-141

[9] Meier R, Tomizaki T, Schulze-Briese C, Baumann U, Stocker A (2003) The molecular basis of vitamin E retention: Structure of human alpha-tocopherol transfer protein. J Mol Biol 331: 725–734. 12899840 10.1016/s0022-2836(03)00724-1

[10] Di Donato I, Bianchi S, Federico A (2010) Ataxia with vitamin E deficiency: update of molecular diagnosis. Neurol Sci 31: 511–515. 20464573 10.1007/s10072-010-0261-1

[11] Muller DPR (2010) Vitamin E and neurological function. Mol Nutr Food Res 54: 710–718. 20183831 10.1002/mnfr.200900460

[12] Dersjant-Li Y, Peisker M (2010) A critical review of methodologies used in determination of relative bio-availability ratio of RRR-alpha-tocopheryl acetate and all-rac-alpha-tocopheryl acetate. J Sci Food Agric 90: 1571–1577. 20564451 10.1002/jsfa.3994

[13] Baumann LS, Md JS (1999) The effects of topical vitamin e on the cosmetic appearance of scars. Dermatologic Surgery 25: 311–315. 10417589 10.1046/j.1524-4725.1999.08223.x

[14] Netscher T (2007) Synthesis of vitamin E. In: Vitamin E: Vitamins and hormones advances in research and applications, 525 B Street, Suite 1900, San Diego, CA 92101–4495 USA: Elsevier Academic Press Inc, volume 76 of *Vitamins and Hormones-Advances in Research and Applications*. 155–202. doi:10.1016/S0083-6729(07)76007-7. 10.1016/S0083-6729(07)76007-7 17628175

[15] Jensen SK, Lauridsen C (2007) alpha-Tocopherol stereoisomers. In: Vitamin E: Vitamins and hormones advances in research and applications, 525 B Street, Suite 1900, San Diego, CA 92101–4495 USA: Elsevier Academic Press Inc, volume 76 of *Vitamins and Hormones-Advances in Research and Applications*. 155–202. doi:10.1016/S0083–6729(07)76007–7. 10.1016/S0083-6729(07)76010-7 17628178

[16] Catignani G (1975) Alpha-tocopherol binding-protein in rat-liver cytoplasm. Biochem Biophys Res Commun 67: 66–72. 1201037 10.1016/0006-291x(75)90283-1

[17] Sato Y, Hagiwara K, Arai H, Inoue K (1991) Purification and characterizations of the alpha-tocopherol transfer protein from rat-liver. FEBS Lett 288: 41–45. 1879562 10.1016/0014-5793(91)80999-j

[18] Aravind L, Neuwald A, Ponting C (1999) Sec14p-like domains in NF1 and Dbl-like proteins indicate lipid regulation of Ras and Rho signaling. Curr Biol 9: R195–R197. 10.1016/s0960-9822(99)80127-4 10209105

[19] Saito K, Tautz L, Mustelin T (2007) The lipid-binding SEC 14 domain. Biochim Biophys Acta-Molecular and Cell Biology of Lipids 1771: 719–726. 10.1016/j.bbalip.2007.02.010 17428729

[20] Kalikin L, Bugeaud E, Palmbos P, Lyons R, Petty E (2001) Genomic characterization of human SEC14L1 splice variants within a 17q25 candidate tumor suppressor gene region and identification of an unrelated embedded expressed sequence tag. Mamm Genome 12: 925–929. 11707779 10.1007/s00335-001-2073-3

[21] Bankaitis VA, Mousley CJ, Schaaf G (2010) The Sec14 superfamily and mechanisms for crosstalk between lipid metabolism and lipid signaling. Trends Biochem Sci 35: 150–160. 19926291 10.1016/j.tibs.2009.10.008 PMC2834860

[22] Panagabko C, Morley S, Hernandez M, Cassolato P, Gordon H, et al (2003) Ligand specificity in the CRAL-TRIO protein family. Biochemistry 42: 6467–6474. 12767229 10.1021/bi034086v

[23] Kaempf-Rotzoll D, Horiguchi M, Hashiguchi K, Aoki J, Tamai H, et al (2003) Human placental trophoblast cells express alpha-tocopherol transfer protein. Placenta 24: 439–444. 12744919 10.1053/plac.2002.0966

[24] Traber M, Arai H (1999) Molecular mechanisms of vitamin E transport. Annu Rev Nutr 19: 343–355. 10448528 10.1146/annurev.nutr.19.1.343

[25] Zhang WX, Thakur V, Lomize A, Pogozheva I, Panagabko C, et al (2011) The Contribution of Surface Residues to Membrane Binding and Ligand Transfer by the alpha-Tocopherol Transfer Protein (alpha-TTP). J Mol Biol 405: 972–988. 21110980 10.1016/j.jmb.2010.11.028 PMC3038628

[26] Brigelius-Flohe R (2006) Bioactivity of vitamin E. Nutr Res Rev. 19: 174–186. 10.1017/S0954422407202938 19079884

[27] Hosomi A, Arita M, Sato Y, Kiyose C, Ueda T, et al (1997) Affinity for alpha-tocopherol transfer protein as a determinant of the biological activities of vitamin E analogs. FEBS Lett 409: 105–108. 9199513 10.1016/s0014-5793(97)00499-7

[28] Min K, Kovall R, Hendrickson W (2003) Crystal structure of human alpha-tocopherol transfer protein bound to its ligand: Implications for ataxia with vitamin E deficiency. Proc Natl Acad Sci U S A 100: 14713–14718. 14657365 10.1073/pnas.2136684100 PMC299775

[29] Campbell S, Stone W, Lee S, Whaley S, Yang H, et al (2006) Comparative effects of RRR-alpha- and RRR-gamma-tocopherol on proliferation and apoptosis in human colon cancer cell lines. BMC Cancer 6: 13. 16417629 10.1186/1471-2407-6-13 PMC1379650

[30] Stocker A (2004) Molecular mechanisms of vitamin E transport Ann N.Y. Acad Sci. 1031: 44–59. 10.1196/annals.1331.005 15753133

[31] Kollman P (1993) Free-Energy calculations - applications to chemical and biochemical phenomena. Chem Rev 93: 2395–2417.

[32] Christ CD, Mark AE, van Gunsteren WF (2010) Basic ingredients of Free Energy calculations: A review. J Comput Chem 31: 1569–1582. 20033914 10.1002/jcc.21450

[33] Oostenbrink C, van Gunsteren W (2005) Free energies of ligand binding for structurally diverse compounds. Proc Natl Acad Sci U S A 102: 6750–6754. 15767587 10.1073/pnas.0407404102 PMC1100734

[34] Zhou R, Das P, Royyuru AK (2008) Single mutation induced H3N2 hemagglutinin antibody neutralization: a Free Energy Perturbation study. J Phys Chem B 112: 15813–15820. 19367871 10.1021/jp805529z

[35] Das P, Li J, Royyuru AK, Zhou R (2009) Free Energy simulations reveal a double mutant avian H5N1 virus hemagglutinin with altered receptor binding specificity. J Comput Chem 30: 1654–1663. 19399777 10.1002/jcc.21274

[36] Schwab F, van Gunsteren WF, Zagrovic B (2008) Computational study of the mechanism and the relative free energies of binding of anticholesteremic inhibitors to squalene-hopene cyclase. Biochemistry 47: 2945–2951. 18247576 10.1021/bi702067h

[37] Zeevaart JG, Wang L, Thakur VV, Leung CS, Tirado-Rives J, et al (2008) Optimization of azoles as anti-human immunodeficiency virus agents guided by free-energy calculations. J Am Chem Soc 130: 9492–9499. 18588301 10.1021/ja8019214 PMC2677907

[38] Reddy M, Erion MD (2001) Calculation of relative binding free energy differences for fructose 1,6-bisphosphatase inhibitors using the thermodynamic cycle perturbation approach. J Am Chem Soc 123: 6246–6252. 11427047 10.1021/ja0103288

[39] Erion MD, Dang Q, Reddy MR, Kasibhatla SR, Huang J, et al (2007) Structure-guided design of AMP mimics that inhibit fructose-1,6-bisphosphatase with high affinity and specificity. J Am Chem Soc 129: 15480–15490. 18041833 10.1021/ja074869u

[40] Helms V, Wade R (1998) Computational alchemy to calculate absolute protein-ligand binding free energy. J Am Chem Soc 120: 2710–2713.

[41] Rastelli G, Thomas B, Kollman P, Santi D (1995) Insight into the specificity of thymidylate synthase from molecular-dynamics and Free-Energy perturbation calculations. J Am Chem Soc 117: 7213–7227.

[42] Brigelius-Flohe R (2009) Vitamin E: The shrew waiting to be tamed. Free Radic Biol Med 46: 543–554. 19133328 10.1016/j.freeradbiomed.2008.12.007

[43] Huang H, Alberg A, Norkus E, Hoffman S, Comstock G, et al (2003) Prospective study of antioxidant micronutrients in the blood and the risk of developing prostate cancer. Am J Epidemiol 157: 335–344. 12578804 10.1093/aje/kwf210

[44] Niesen FH, Berglund H, Vedadi M (2007) The use of differential scanning fluorimetry to detect ligand interactions that promote protein stability. Nature Protocols 2: 2212–2221. 17853878 10.1038/nprot.2007.321

[45] Layton CJ, Hellinga HW (2010) Thermodynamic Analysis of Ligand-Induced Changes in Protein Thermal Unfolding Applied to High-Throughput Determination of Ligand Affinities with Extrinsic Fluorescent Dyes. Biochemistry 49: 10831–10841. 21050007 10.1021/bi101414z

[46] Pace CN, Laurents DV (1989) A new method for determining the heat capacity change for protein folding. Biochemistry 28: 2520–2525. 2499351 10.1021/bi00432a026

[47] Holdgate GA, Ward WH (2005) Measurements of binding thermodynamics in drug discovery. Drug Discov. Today 10: 1543–1550. 10.1016/S1359-6446(05)03610-X 16257377

[48] Akutagawa S (1995) Asymmetric synthesis by metal binap catalysts. Appl Cat A: General 128: 171–207.

[49] Wang X, Thomas B, Sachdeva R, Arterburn L, Frye L, et al (2006) Mechanism of arylating quinone toxicity involving Michael adduct formation and induction of endoplasmic reticulum stress. Proc Natl Acad Sci U S A 103: 3604–3609. 16505371 10.1073/pnas.0510962103 PMC1450130

[50] Scheuner D, Mierde D, Song B, Flamez D, Creemers J, et al (2005) Control of mRNA translation preserves endoplasmic reticulum function in beta cells and maintains glucose homeostasis. Nat Med 11: 757–764. 15980866 10.1038/nm1259

[51] Ryu EJ, Harding HP, Angelastro JM, Vitolo OV, Ron D, et al (2002) Endoplasmic reticulum stress and the unfolded protein response in cellular models of parkinson's disease. J Neurosci 22: 10690–10698. 12486162 10.1523/JNEUROSCI.22-24-10690.2002 PMC6758450

[52] Nakagawa T, Zhu H, Morishima N, Li E, Xu J, et al (2000) Caspase-12 mediates endoplasmic-reticulum-specific apoptosis and cytotoxicity by amyloid-[beta]. Nature 403: 98–103. 10638761 10.1038/47513

[53] Hornak V, Abel R, Okur A, Strockbine B, Roitberg A, et al (2006) Comparison of multiple amber force fields and development of improved protein backbone parameters. Proteins Struct Funct Bioinf 65: 712–725. 10.1002/prot.21123 PMC4805110 16981200

[54] Wang J, Wolf R, Caldwell J, Kollman P, Case D (2004) Development and testing of a general amber force field. J Comput Chem 25: 1157–1174. 15116359 10.1002/jcc.20035

[55] Cornell W, Cieplak P, Bayly C, Gould I, Merz K, et al (1995) A 2nd generation force-field for the simulation of proteins, nucleic-acids, and organic-molecules. J Am Chem Soc 117: 5179–5197.

[56] Stephens PJ, Devlin FJ, Chabalowski CF, Frisch MJ (1994) Ab initio calculation of vibrational absorption and circular dichroism spectra using density functional force fields. J Phys Chem 98: 11623–11627.

[57] Frisch MJ, Trucks GW, Schlegel HB, Scuseria GE, Robb MA, et al. Gaussian 03, Revision C.02.

[58] Jorgensen W, Chandrasekhar J, Madura J, Impey R, Klein M (1983) Comparison of simple potential functions for simulating liquid water. J Chem Phys 79: 926–935.

[59] Nose S (1984) A molecular-dynamics method for simulating in the canonical ensemble. Mol Phys 52: 255–268.

[60] Hoover W (1985) Canonical dynamics- equilibrium phase-space distributions. Phys Rev A 31: 1695–1697. 10.1103/physreva.31.1695 9895674

[61] Martyna G, Klein M, Tuckerman M (1992) Nose-Hoover chains - the canonical ensemble via continuous dynamics. J Chem Phys 97: 2635–2643.

[62] Parrinello M, Rahman A (1981) Polymorphic transitions in single-crystals - a new molecular-dynamics method. J Appl Phys 52: 7182–7190.

[63] Essmann U, Perera L, Berkowitz M, Darden T, Lee H, et al (1995) A smooth particle mesh ewald method. J Chem Phys 103: 8577–8593.

[64] Hess B, Bekker H, Berendsen H, Fraaije J (1997) LINCS: A linear constraint solver for molecular simulations. J Comput Chem 18: 1463–1472.

[65] Miyamoto S, Kollman P (1992) SETTLE - An analytical version of the shake and rattle algorithm for rigid water models. J Comput Chem 13: 952–962.

[66] Beutler T, Mark A, Vanschaik R, Gerber P, van Gunsteren W (1994) Avoiding singularities and numerical instabilities in Free-Energy calculations based on molecular simulations. Chem Phys Lett 222: 529–539.

[67] van Gunsteren W, Mark A (1998) Validation of molecular dynamics simulation. J Chem Phys 108: 6109–6116.

[68] Byrd R, Lu P, Nocedal J, Zhu C (1995) A limited memory algorithm for bound constrained optimization. Siam J Sci Comput 16: 1190–1208.

[69] Zhu C, Byrd R, Pehuang Lu, Nocedal J (1997) L-BFGS-B: Fortran subroutines for large-scale bound-constrained optimization. ACM T Math Software 23: 550–60.

[70] Humphrey W, Dalke A, Schulten K (1996) VMD – Visual Molecular Dynamics. J Mol Graphics 14: 33–38. 10.1016/0263-7855(96)00018-5 8744570

[71] Van der Spoel D, Lindahl E, Hess B, Groenhof G, Mark A, et al (2005) GROMACS: Fast, flexible, and free. J Comput Chem 26: 1701–1718. 16211538 10.1002/jcc.20291

[72] Berendsen H, van der Spoel D, van Drunen R (1995) GROMACS - a message-passing parallel molecular-dynamics implementations. Comput Phys Commun 91: 43–56.

[73] Lindahl E, Hess B, van der Spoel D (2001) GROMACS 3.0: a package for molecular simulation and trajectory analysis. J Mol Model 7: 306–317.

[74] Hess B, Kutzner C, van der Spoel D, Lindahl E (2008) GROMACS 4: Algorithms for highly efficient, load-balanced, and scalable molecular simulation. J Chem Theory Comput 4: 435–447. 26620784 10.1021/ct700301q

