# Supplementary figures and images for: Engineering Tocopherol Selectivity in α-TTP: A Combined In Vitro/In Silico Study
Source: PLoS One. 2012 Nov 13;7(11):e49195. doi: 10.1371/journal.pone.0049195 (PMC3496730; doi:10.1371/journal.pone.0049195)

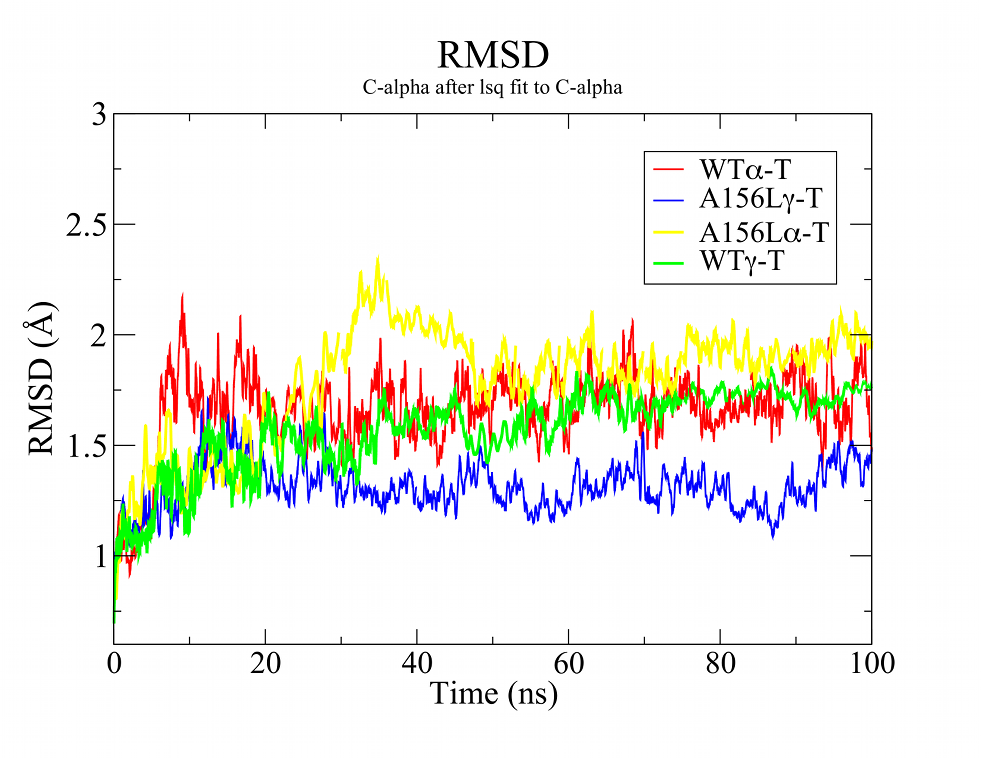

Supplement: Figure S1 — RMSD of the C atoms of the different complexes during the 100 ns of MD simulations. (TIFF) [file pone.0049195.s001.tiff]

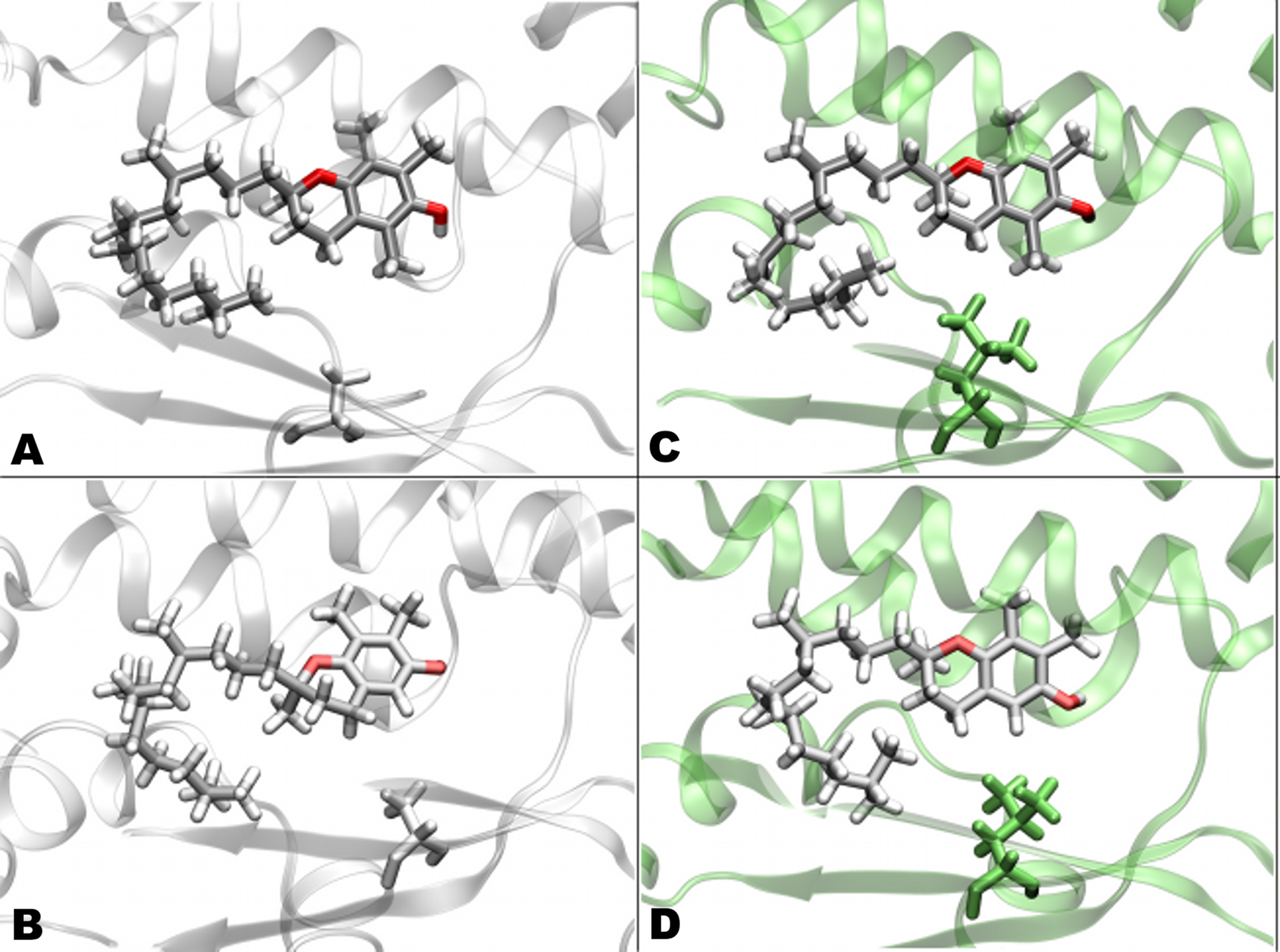

Supplement: Figure S2 — Average structures of topocherol in the studied complexes. Panel A: WT-T; Panel B: WT-T; Panel C: A156L-T; Panel D: A156L-T. (TIFF) [file pone.0049195.s002.tiff]

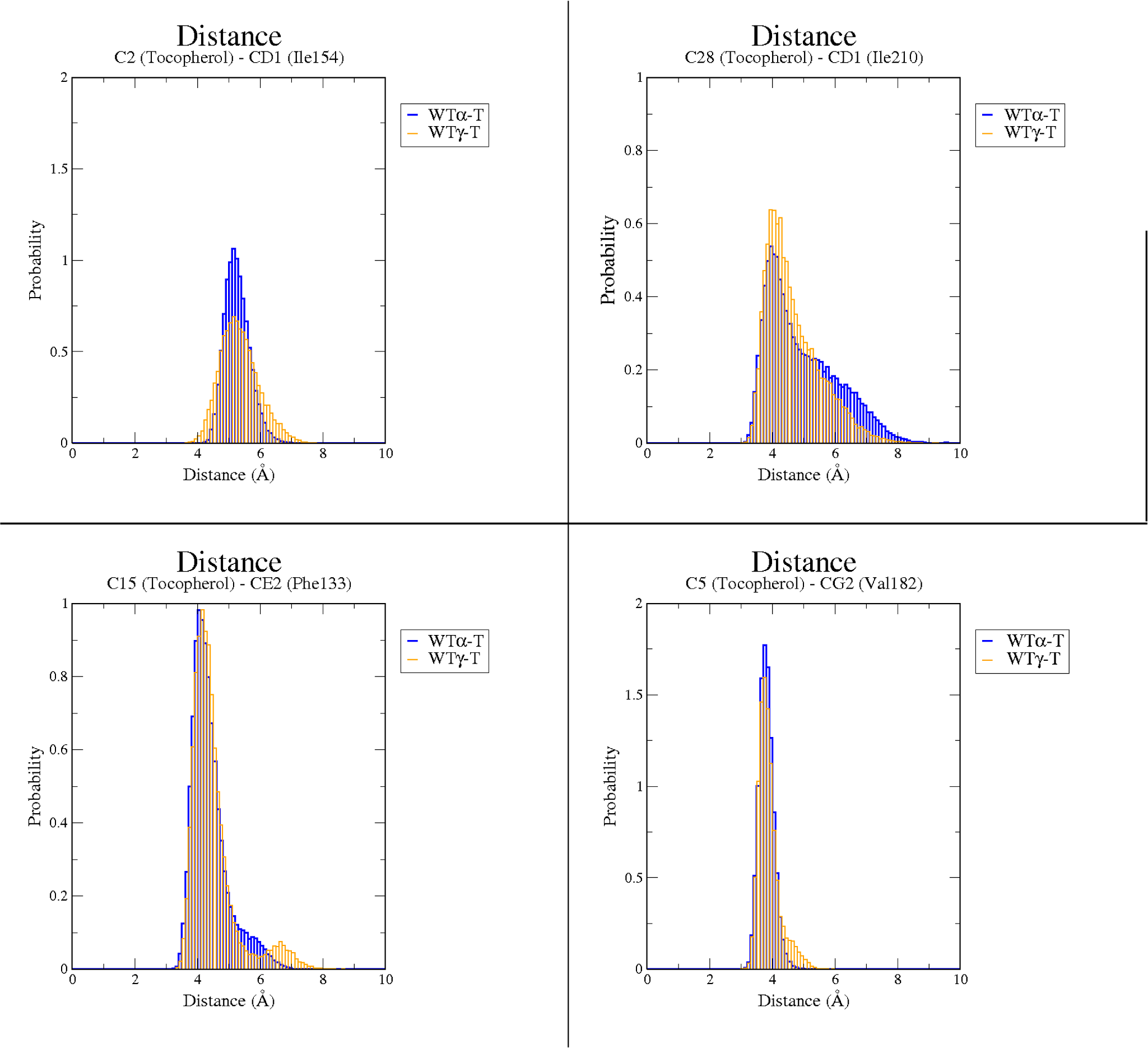

Supplement: Figure S3 — Statistical distribution of selected interatomic distances between tocopherol and residues in the binding pocket for the WT -T and WT -T complexes. (TIFF) [file pone.0049195.s003.tiff]

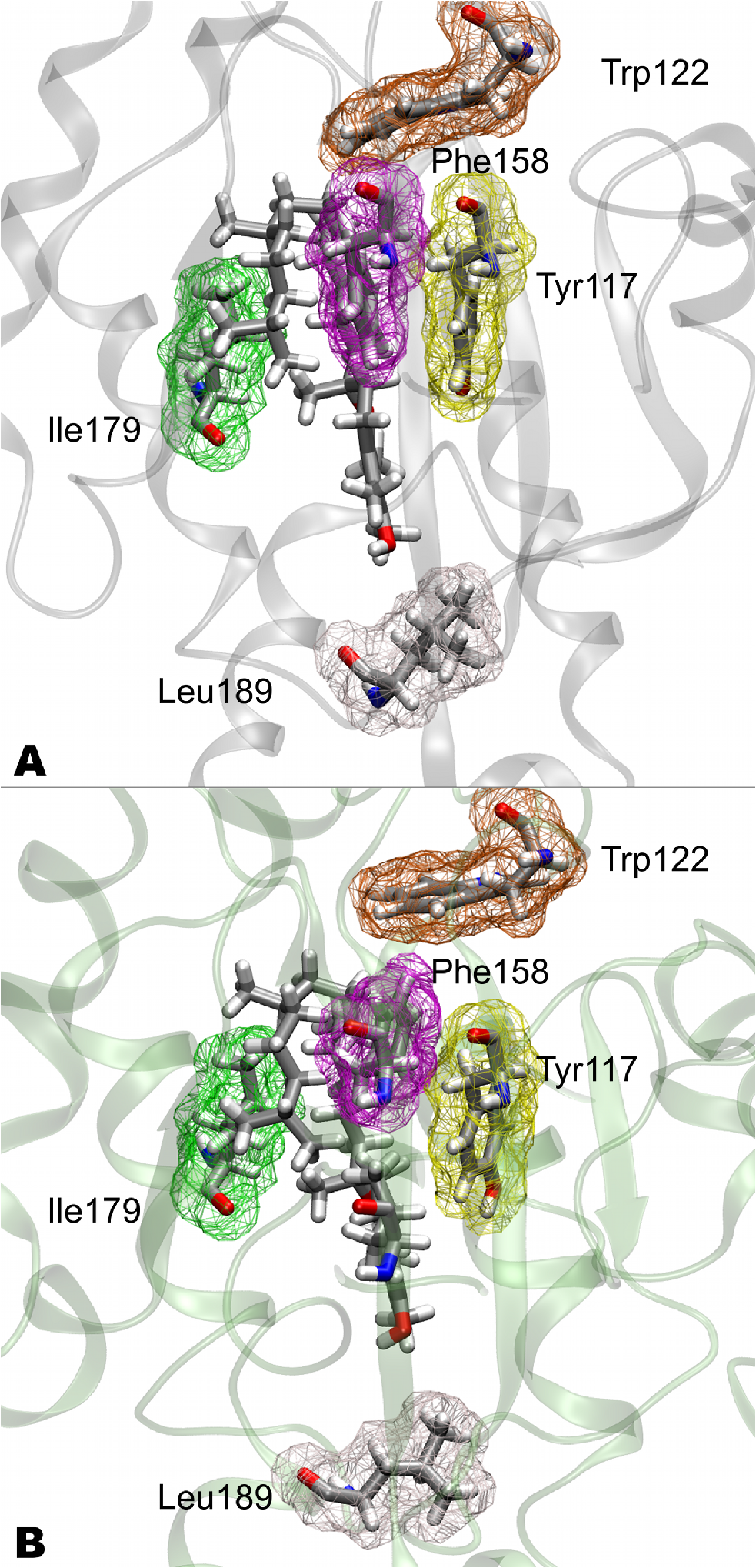

Supplement: Figure S4 — Ligand-protein hydrophobic contacts in WT -T (panel A) and 156L -T (panel B). The van der Waals space of residues in contact with tocopherol is highlighted by wireframe representation. (TIFF) [file pone.0049195.s004.tiff]

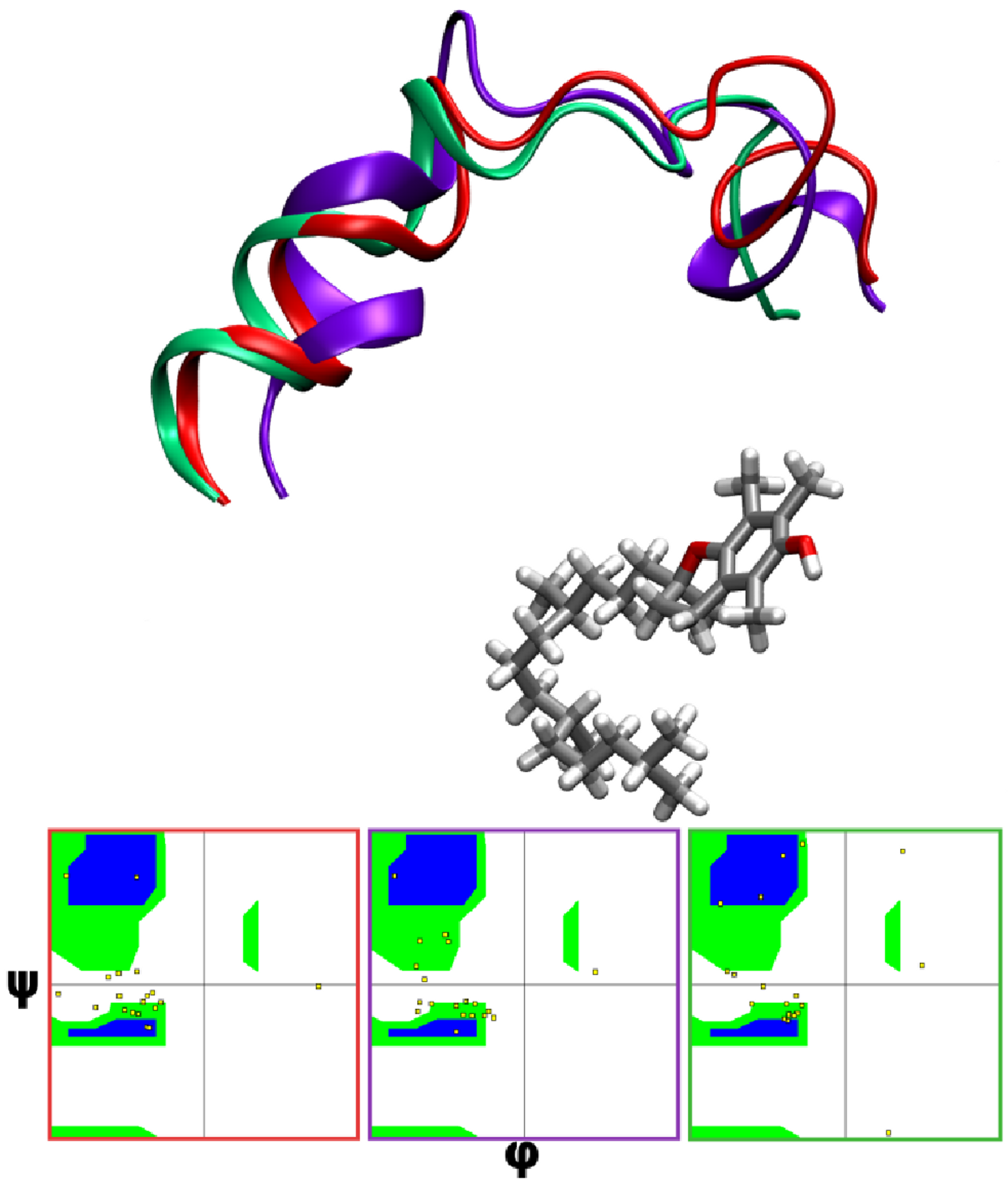

Supplement: Figure S5 — Comparison of the structure of the H4-H5 segment for WT -T (in red), WT -T (violet) and A156L -T (green) complexes. The respective distribution of the amino acids in the Ramachandran plot is shown in the bottom panels. (TIFF) [file pone.0049195.s005.tiff]

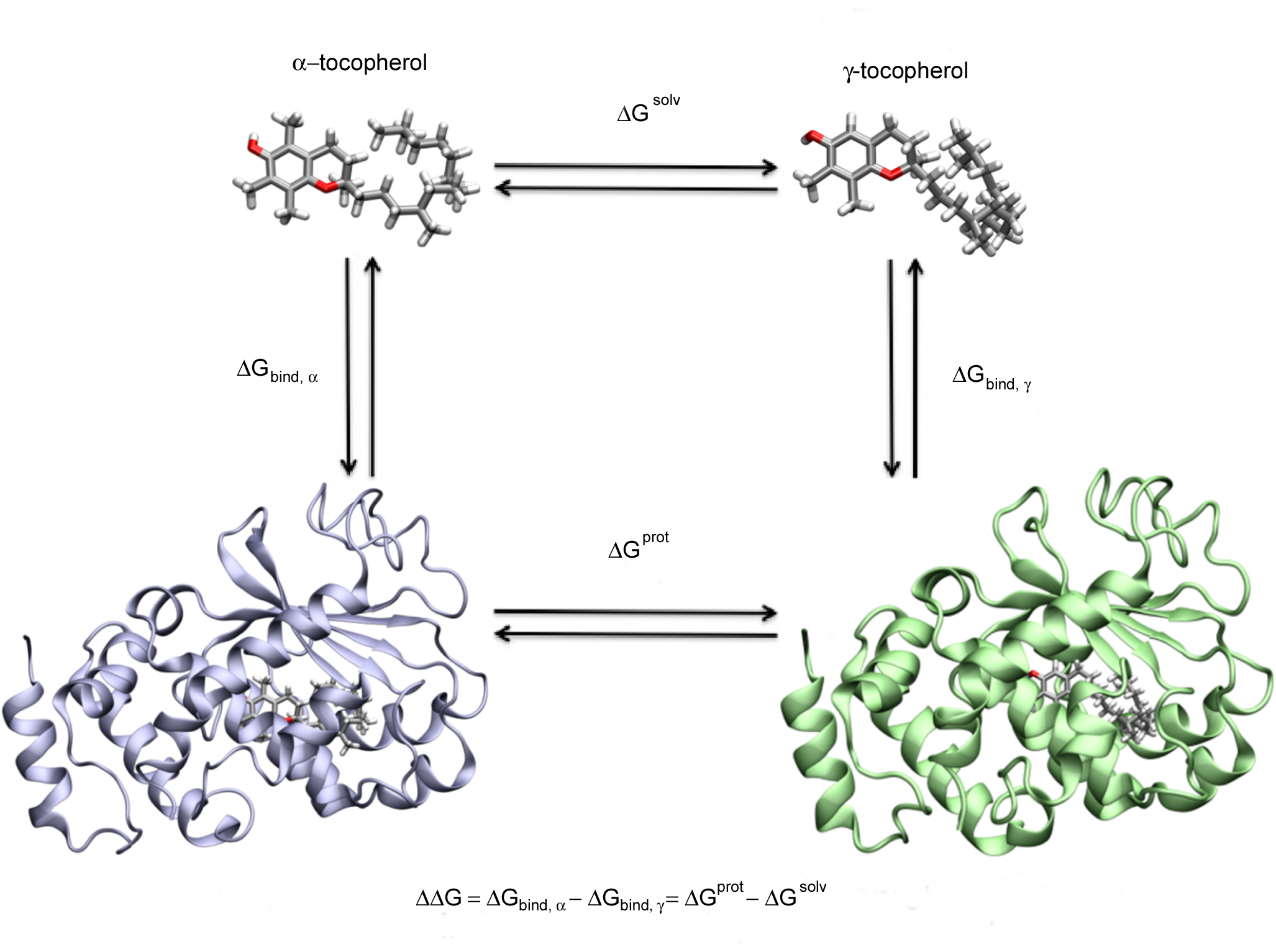

Supplement: Figure S6 — Scheme of the thermodynamic cycle used to compute the relative binding affinity of -T and -T to TTP. Free-energy perturbation is used to estimate and . (TIFF) [file pone.0049195.s006.tiff]

| Dihedral Angle | WT $\alpha$ -T | WT $\gamma$ -T | A156L $\alpha$ -T | A156L $\gamma$ -T |
|----------------|----------------|----------------|-------------------|-------------------|
| $\phi$ 1       | 176.62         | -169.54        | 166.72            | -179.43           |
| $\phi$ 2       | 49.70          | 55.04          | 159.63            | 45.56             |
| $\phi$ 3       | 177.20         | 175.46         | -78.27            | 172.90            |

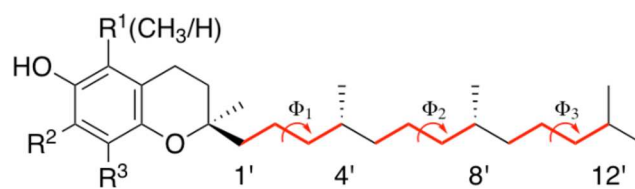

Supplement: Table S2 — Comparison between specific dihedral angles of the hydrophobic tail of tocopherol in differerent tocopherol-TTP complexes. The dihedral angles under consideration are highlighted in the bottom scheme. (PDF) [file pone.0049195.s008.pdf]
