# Supplementary material for: Engineering Tocopherol Selectivity in α-TTP: A Combined In Vitro/In Silico Study
Source: PLoS One. 2012 Nov 13;7(11):e49195. doi: 10.1371/journal.pone.0049195 (PMC3496730; doi:10.1371/journal.pone.0049195)
Supplement: Table S1 — Average H-bonding distances (in Å ) between the chromanol hydroxyl group of tocopherol and surrounding partners in each system. (PDF) [file pone.0049195.s007.pdf]

H-bond	WTα-T	WTγ-T	A156Lα-T	A156Lγ-T
(Vit)-O $\cdots$ HO-(Ser140)	2.17	2.05	1.79	(4.89)
(Vit)-OH $\cdots$ OH_2	1.87	1.92	1.64	1.79
