# Supplementary material for: Engineering Tocopherol Selectivity in α-TTP: A Combined In Vitro/In Silico Study
Source: PLoS One. 2012 Nov 13;7(11):e49195. doi: 10.1371/journal.pone.0049195 (PMC3496730; doi:10.1371/journal.pone.0049195)
Supplement: Table S3 — Comparison between average values of the side-chain dihedral angles of residues in the binding pocket (top part) and relevant residues of the lid (bottom part) for the different complexes under study. The dihedral angle is the one corresponding to the rotation aroun the bond; the dihedral angle is defined by the bond. (PDF) [file pone.0049195.s009.pdf]

Residue		WTα	WTγ	A156Lα	A156Lγ
Phe133	χ_1	-107.63	-173.56	-151.56	-147.30
	χ_2	129.56	-166.11	-0.29	-46.10
Val182	χ_1	-62.22	-66.24	-65.74	-65.78
Leu183	χ_1	-52.67	-94.31	-68.02	-150.29
	χ_2	-176.19	50.49	-177.101	60.33
Val206	χ_1	-58.09	-62.88	177.41	79.64
Phe207	χ_1	179.37	171.37	177.50	-172.55
	χ_2	-114.61	-103.27	74.77	79.29
Ile210	χ_1	71.78	-167.53	170.46	-53.01
	χ_2	166.37	162.72	55.83	166.28
